# *Calendula* L. (Asteraceae) specimens held at AVE (Herbarium of the University of Aveiro)

**DOI:** 10.3897/BDJ.14.e211502

**Published:** 2026-11-02

**Authors:** Paulo Silveira

**Affiliations:** 1 Department of Biology & CESAM, University of Aveiro, Aveiro, Portugal Department of Biology & CESAM, University of Aveiro Aveiro Portugal

**Keywords:** Algeria, *
Calendula
*, Canary Islands, Lebanon, Morocco, occurrence dataset, Portugal, Spain, Tunisia

## Abstract

**Background:**

Recently, P. Silveira et al. published partial taxonomic revisions of the genus *Calendula*, covering the Iberian Peninsula, Morocco, the Canary Islands and some taxa from Algeria and Tunisia. In these studies, achene morphology and genome size estimates were considered particularly relevant for taxonomic purposes, alongside chromosome number, leaf and flower/capitula morphology and life span. The taxonomic concept previously proposed by C. Heyn et al. for the annual plants with 44 chromosomes was adopted, but a mixed approach was preferred for the perennial plants with 32 chromosomes: most of the subspecies recognised by H. Ohle were adopted, but all were placed under *C. suffruticosa* rather than split between this taxon and *C. incana*. This approach was preferred because a gradient of morphological variation and DNA content was observed amongst the plants that H. Ohle had included in the two species, rather than a clear and consistent separation between the two groups. Furthermore, O. Plume, who studied the genus using molecular markers, found no evidence of closer phylogenetic relationships amongst the taxa within these two species. Overall, these recent taxonomic revisions expanded and consolidated knowledge of the genus' taxonomy and diversity.

**New information:**

The dataset includes *Calendula* specimens held at AVE (Herbarium of the University of Aveiro). It comprises 406 occurrence records (specimen metadata), together with images of 77 specimens, which were used in the recent taxonomic revisions by P. Silveira et al., eight of which being type specimens. Data for most of these specimens had already been published in those papers, but in a more abbreviated form, lacking the completeness of the Darwin Core format adopted here, which facilitates use by other researchers. Most of the specimens were collected by P. Silveira and his team, but numerous donated specimens are disclosed here for the first time, such as those received from BH herbarium that were collected by O. Plume in regions not sampled by P. Silveira, like Sicily (Italy) and Lebanon, which were also used during her PhD research. In addition, the dataset contains many duplicates from the HAL herbarium, collected by H. Ecklerlein, H. Meusel, H. Ohle and K.H. Rechinger and used by H. Ohle in his taxonomic revisions of the perennial *Calendula* taxa and which have not been cited in our previous publications.

## Introduction

The genus *Calendula* L. (Calenduleae, Asteraceae) has been considered taxonomically difficult (Norlindh 1977, Heyn and Joel 1983). It was reported to include 10-27 species, depending on the taxonomic concept followed. During the 20^th^ century, the two most important taxonomic revisions were conducted by Heyn et al. (1974) for the annual species and by H. Ohle (Ohle 1974, Ohle 1975a, Ohle 1975b) for the perennial ones. However, no consensus was reached on which characters should be used to distinguish taxa and different taxonomic treatments continued to appear, like in the *Flora Europaea* (Meikle 1976), which disregarded several species recognised by the previous cited work by H. Ohle.

More recently, P. Silveira and his team published partial taxonomic revisions for the Iberian Peninsula, Morocco and some taxa from Algeria and Tunisia and the Canary Islands (Silveira et al. 2013, Gonçalves et al. 2018, Gonçalves et al. 2023, Simão et al. 2024), describing four new species and nine new subspecies, which, along with four new combinations, settled the number of taxa to 17 species, one of them, *C. suffruticosa* Vahl, with 27 subspecies.

The purpose of this paper is data mobilisation for the specimens kept at AVE (Herbarium of the University of Aveiro) and used for these taxonomic revisions, to be made available on GBIF (https://www.gbif.org/). Most of the specimens were collected by P. Silveira and his team, but a significant part, like those collected by O. Plume on the scope of her PhD (Plume 2015) and a set of specimens from HAL, were donated to AVE.

## General description

### Purpose

This dataset was produced in the course of partial taxonomical revisions of the genus *Calendula* in the Iberian Peninsula, Morocco (with some taxa from Algeria and Tunisia) and the Canary Islands. It is available on GBIF (https://www.gbif.org) so that it can be consulted and used by any interested researcher and includes 77 images, eight of them of type specimens.

## Project description

### Title

*Calendula* specimens held at AVE (Herbarium of the University of Aveiro)

### Personnel

Paulo C. da Silveira

### Study area description

Mostly Iberian Peninsula, Macaronesia and the understudied north of Africa (Morocco, Algeria and Tunisia).

### Design description

The processing of the *Calendula* backlog included: mounting of the specimens on cardboard, including labels and using appropriate gummed archival linen tape. Type specimens were wrapped in their appropriate red banded sheets, while all the other specimens were enclosed in parchment paper and genus folders. This renders these collections ready to be consulted by any researcher upon request. Furthermore, their metadata, which has already been databased, is made available on GBIF (https://www.gbif.org) along with a selection of 77 images, which include eight type specimens.

### Funding

IAPT 2025 “Small Collections Grant”.

## Sampling methods

### Study extent

These data cover only a small part of the AVE collection, dedicated to the genus *Calendula* L. Geographically, it encompasses specimens from Spain (182), Portugal (83), Morocco (77), Italy (23, all from Sicily), Lebanon (17), Algeria (13), Tunisia (6), Israel (1), Palestine (1) and Greece (1). The temporal boundaries ranges within 1966-1981 for collections of German botanists, which were donated by HAL to AVE and 30 April 2022 collections made in the course of the expeditions to the Canary Islands made by P. Silveira's team (including I. Simão and J.A. Reyes-Betancort).

### Sampling description

Sampling of the specimens was done following the usual methods described for their collection, preparation and mounting (Pinho et al. 2016).

### Quality control

Consistency and standardisation of the geography, people's names, taxonomy, dates etc. were reviewed using BRAHMS 7.6 (Oxford University, UK) tools. Coordinate uncertainty of the specimens that did not include precise coordinates of the collection site was estimated using Geopick (https://geopick.gbif.org).

### Step description

The collection was databased in BRAHMS 7.6 (Oxford University, UK), either directly from the field books (team's collections) or from original specimens' labels/documentation (other collector's specimens offered to AVE). Taxonomy fields, including authorship names, were checked against IPNI (https://www.ipni.org/) and POWO (https://powo.science.kew.org/). Standardisation and checking of consistency was done with the tools available in BRAHMS 7.6. A selection of specimens, at least one for each taxon, several for more common and morphologically diverse taxa, were imaged using a digital camera Canon 100D, installed inside a Godox DF-01 Light cube 80 × 80 × 80 cm and connected to a PC running digiCamControl 2.1.6.0 (2018 Duka István). Contrast and light were adjusted using Paint.NET 5.1.12 (dotPDN LLC) and images were saved in JPG format and deposited at https://archive.org/.

## Geographic coverage

### Description

These specimens are mostly from continental Portugal, Spain and Morocco, but with scattered specimens collected from the west in the Azores to the east in the Lebanon and from the north in Galicia and Greece to the south in the Canary Islands and south Morocco from latitudes of approx. 28 to 43 degrees and from longitudes of -25 to 36 degrees. Some specimens were cultivated outside their original range.

### Coordinates

27.861389 and 42.638611 Latitude; -25.017323 and 36.540012 Longitude.

## Taxonomic coverage

### Description

This dataset pertains only to the genus *Calendula* L., belonging to the family Asteraceae.

### Taxa included

**Table:** 

Rank	Scientific Name	
genus	* Calendula *	

## Temporal coverage

### Notes

28-04-1966 through 30-04-2022.

## Usage licence

### Usage licence

Other

### IP rights notes

This work is licensed under a Creative Commons Attribution (CC-BY 4.0) License.

## Data resources

### Data package title

*Calendula* L. specimens held at AVE (Herbarium of the University of Aveiro)

### Resource link

https://doi.org/10.15468/sqkv45

### Alternative identifiers

https://ipt.gbif.pt/ipt/resource?r=calendula_specimens_at_ave&v=1.9

### Number of data sets

1

### Data set 1.

#### Data set name

*Calendula* specimens held at AVE (Herbarium of the University of Aveiro)

#### Data format

Darwin Core

#### Description

This dataset includes *Calendula* specimens held at AVE (Herbarium of the University of Aveiro) (Silveira 2026) and used for the recently published partial taxonomic revisions for the Iberian Peninsula, Morocco and some taxa from Algeria and Tunisia and the Canary Islands, by P. Silveira et al. (Gonçalves et al. 2018; Gonçalves et al. 2023; Simão et al. 2024). Most of the specimens were collected by P. Silveira and collaborators, but some collections were donated. For example, the latter include specimens collected and cultivated by Olofron Plume, in the course of her PhD at Cornell University (BH), and duplicates from HAL (Martin-Luther-Universität Halle-Wittenberg Herbarium).

**Data set 1.** 

Column label	Column description
occurrenceID	A persistent global unique identifier, generated for each occurrence.
datasetName	Name of the source dataset.
basisOfRecord	The specific nature of the data record. A Preserved specimen in all cases.
recordedBy	Name for the collector(s) of the specimen, responsible for recording each dwc:Occurrence.
recordNumber	Specimen collector's number attributed in the P.Silveira's collection.
otherCatalogNumbers	Other specimen collector's number.
institutionCode	Acronym of the Herbarium of the University of Aveiro attributed in Index Herbariorum.
catalogNumber	An unique identifier atributed for each specimen within the Herbarium of the University of Aveiro collection.
family	The full scientific name of the family in which the dwc:Taxon is classified.
genus	The full scientific name of the genus in which the dwc:Taxon is classified.
specificEpithet	The name of the first or species epithet of the dwc:scientificName.
verbatimTaxonRank	The taxonomic rank of the most specific name in the dwc:scientificName as it appears in the original record.
infraspecificEpithet	The name of the lowest or terminal infraspecific of the dwc:scientificName.
scientificNameAuthorship	The authorship information for the dwc:scientificName formatted according to the conventions of the applicable dwc:nomenclaturalCode.
scientificName	The full scientific name, including authorship.
verbatimIdentification	A string representing the taxonomic identification as it appeared in the original record.
country	The name of the country or major administrative unit in which the dcterms:Location occurs.
islandGroup	The name of the island group in which the dcterms:Location occurs.
stateProvince	The name of the next smaller administrative region than country (state, province, canton, department, region etc.) in which the dcterms:Location occurs.
municipality	The full, unabbreviated name of the next smaller administrative region than county (city, municipality etc.) in which the dcterms:Location occurs.
verbatimLocality	An original textual description of a dcterms:Location.
verbatimElevation	An original description of the elevation of a dcterms:Location.
maximumElevationInMetres	The greatest elevation within a range of elevations, measured relative to the vertical reference surface.
habitat	A category or description of the habitat.
fieldNotes	Notes taken in the field about the dwc:Occurrence.
day	The integer day of the month on which the specimen was collected.
month	The integer month in which the specimen was collected.
year	The four-digit year in which the specimen was collected, according to the Common Era Calendar.
eventDate	A date-time or time interval during which the specimen was collected.
dateIdentified	The date on which the specimen was determined as representing the dwc:Taxon.
identifiedBy	Name of the expert responsible for making the identification of the specimen.
decimalLatitude	A geographic latitude (in decimal degrees, using the spatial reference system given in dwc:geodeticDatum) of a dcterms:Location.
decimalLongitude	A geographic longitude (in decimal degrees, using the spatial reference system given in dwc:geodeticDatum) of a dcterms:Location.
associatedMedia	A list (concatenated and separated) of identifiers (publication, global unique identifier, URI) of media associated with the dwc:Occurrence.
type	The nature or genre of the resource.
typeStatus	A list (concatenated and separated) of nomenclatural types (type status, typified scientific name, publication) applied to the subject.
typifiedName	A scientific name for which a specimen or other name is the type.
licence	A legal document giving official permission to do something with the resource.
locationID	An identifier for a dcterms:Location.
locality	The specific description of the place.
geodeticDatum	The ellipsoid, geodetic datum or spatial reference system (SRS), upon which the geographic coordinates given in dwc:decimalLatitude and dwc:decimalLongitude are based.
coordinateUncertaintyInMetres	A horizontal distance (in metres) from a given dwc:decimalLatitude and dwc:decimalLongitude describing the smallest circle containing the whole of the dcterms:Location.
coordinatePrecision	A decimal representation of the precision of the coordinates given in the dwc:decimalLatitude and dwc:decimalLongitude.
pointRadiusSpatialFit	A ratio of the area of a point-radius (dwc:decimalLatitude, dwc:decimalLongitude, dwc:coordinateUncertaintyInMetres) to the area of a true (original or most specific) spatial representation of a dcterms:Location.
footprintWKT	A Well-Known Text (WKT) representation of the shape (footprint, geometry) that defines a dcterms:Location.
footprintSRS	The ellipsoid, geodetic datum or spatial reference system (SRS), upon which the geometry given in dwc:footprintWKT is based.
footprintSpatialFit	A ratio of the area of a footprint (dwc:footprintWKT) to the area of a true (original or most specific) spatial representation of a dcterms:Location.
georeferencedBy	A name for a dcterms:Agent responsible for providing a georeference.
georeferencedDate	The date on which the dcterms:Location was georeferenced.
georeferenceProtocol	A description or reference to a dwc:Protocol used to determine a spatial footprint, coordinates and uncertainties.
georeferenceSources	A list (concatenated and separated) of maps, gazetteers or other resources used to georeference a dcterms:Location, described specifically enough to allow anyone in the future to use the same resources.
georeferenceRemarks	Comments or notes about the spatial description determination, explaining assumptions made in addition or opposition to the those formalised in the method referred to in dwc:georeferenceProtocol.
kingdom	The full scientific name of the kingdom in which the dwc:Taxon is classified.
taxonRank	The taxonomic rank of the most specific name in the dwc:scientificName.
countryCode	The standard code (ISO 3166-1-alpha-2) for the country in which the dcterms:Location occurs.
